# FEASIBILITY AND ACCEPTABILITY OF IMPLEMENTING DEPRESCRIBING IN POST-ACUTE HOME HEALTH CARE

**DOI:** 10.1093/geroni/igae098.4265

**Published:** 2024-12-31

**Authors:** Jinjiao Wang, Jenny Shen, Fang Yu, Kobi Nathan, Yeates Conwell, Sandra Simmons, Amanda Mixon, Thomas Caprio

**Affiliations:** University of Rochester - School of Nursing, Rochester, New York, United States; University of Rochester Medical Center, Rochester, New York, United States; Arizona State University, Phoenix, Arizona, United States; Saint John Fisher University, Rochester, New York, United States; University of Rochester Medical Center, Rochester, New York, United States; Vanderbilt University Medical Center, Nashville, Tennessee, United States; Vanderbilt University Medical Center, Nashville, Tennessee, United States; University of Rochester Medical Center, Rochester, New York, United States

## Abstract

Polypharmacy is common in home health care (HHC), but no deprescribing intervention has been tested in HHC. In a prior study, we identified essential components of deprescribing by interviewing patients, primary, acute and post-acute care providers, pharmacists, and HHC nurses in 12 U.S. states. This study assessed the feasibility and acceptability of deprescribing in HHC through the collaboration of an HHC nurse and a pharmacist to conduct home medication review, deprescribing assessment, primary care provider discussion, patient education, deprescribing implementation, and safety monitoring. In 11 patients who received HHC following hospital discharge, we assessed feasibility and acceptability of deprescribing through surveys and qualitative interviews with patients, providers, and study interventionists. Patients received deprescribing components over 3-4 home visits. On average, the HHC nurse spent 1 hour/home visit/patient, the pharmacist spent 1 hour on deprescribing assessment/patient, and the provider spent 5 minutes / patient to review and implement deprescribing recommendations. Over 70% of deprescribing recommendations were accepted by providers, with 30% not accepted mostly due to patient preference. Providers found deprescribing “very feasible,” but noted barriers such as time constraints and patient preferences. The HHC nurse found deprescribing components “very feasible,” citing the multidisciplinary approach as a facilitator but noting communication challenges with providers. Providers and patients both generally reported that deprescribing components were helpful and led to patients feeling empowered. In conclusion, deprescribing components were feasible in HHC and highly acceptable to patients and providers. Effective communication between patients, HHC nurses, and providers is crucial for deprescribing in HHC.

